# Supplementary figures and images for: Kaempferol as an Alternative Cryosupplement for Bovine Spermatozoa: Cytoprotective and Membrane-Stabilizing Effects
Source: Int J Mol Sci. 2024 Apr 8;25(7):4129. doi: 10.3390/ijms25074129 (PMC11012659; doi:10.3390/ijms25074129)

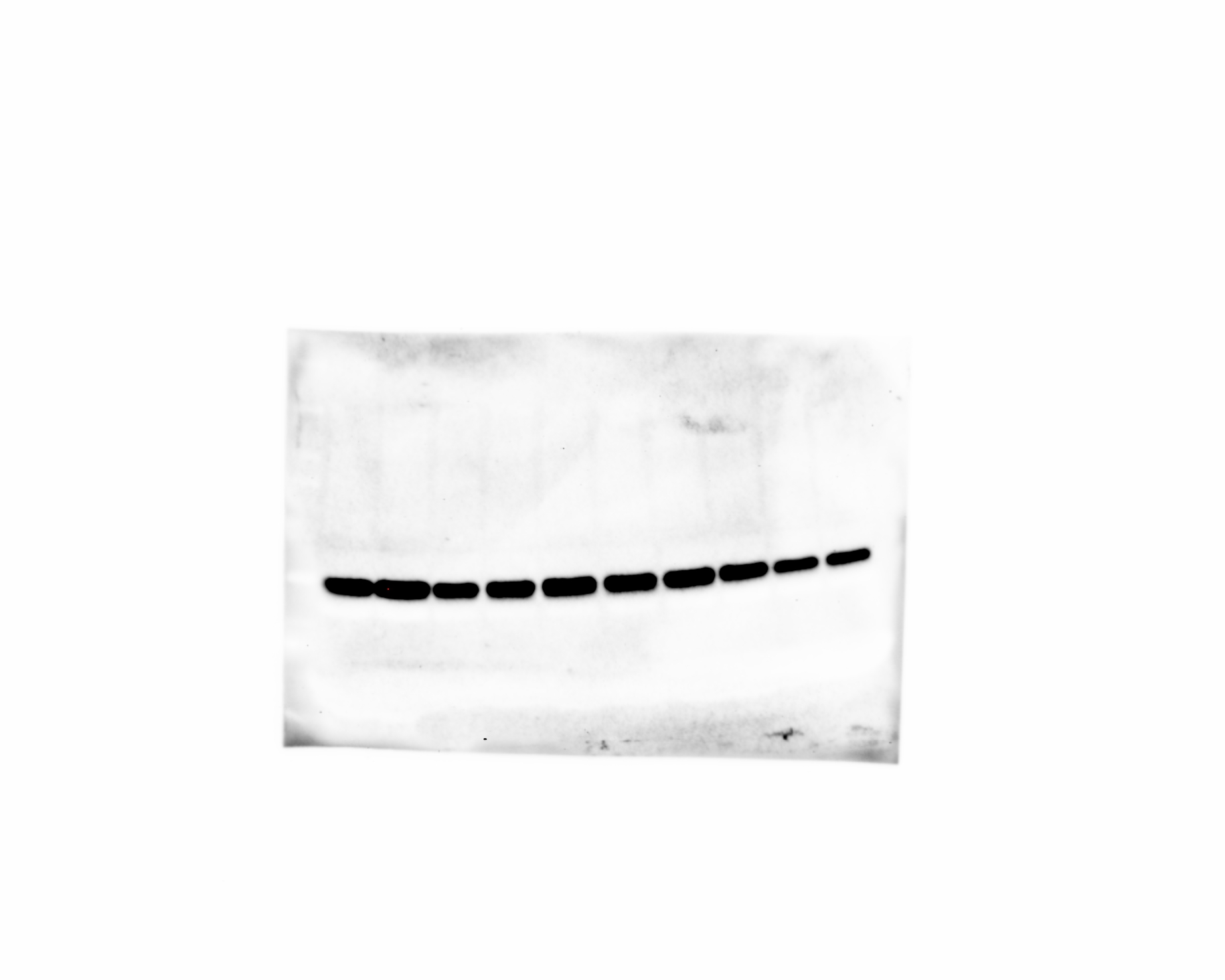

Supplement: Supplementary file 1 [file ijms-25-04129-s001.zip › IJMS WB/PKA.jpg]

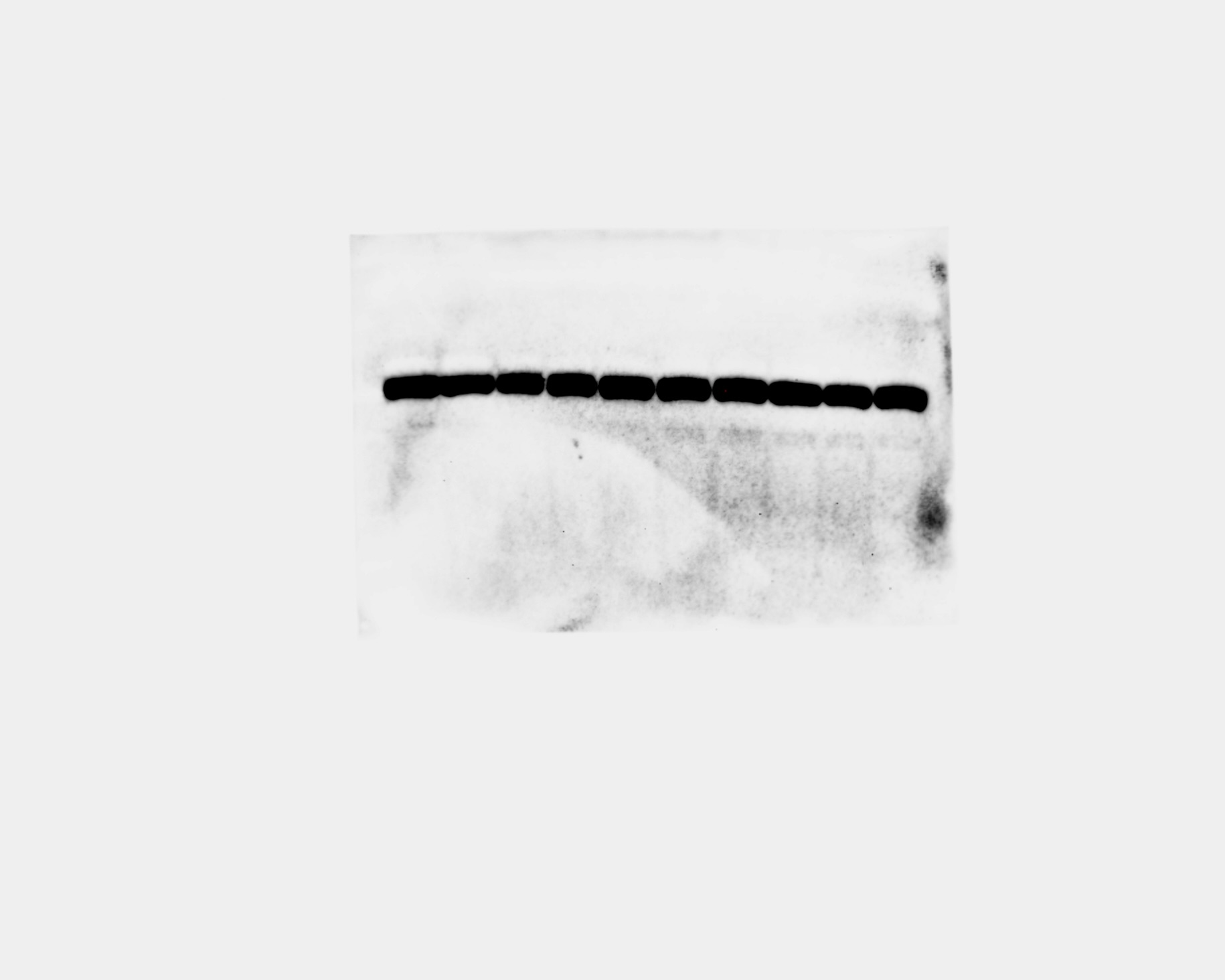

Supplement: Supplementary file 1 [file ijms-25-04129-s001.zip › IJMS WB/PKC.jpg]

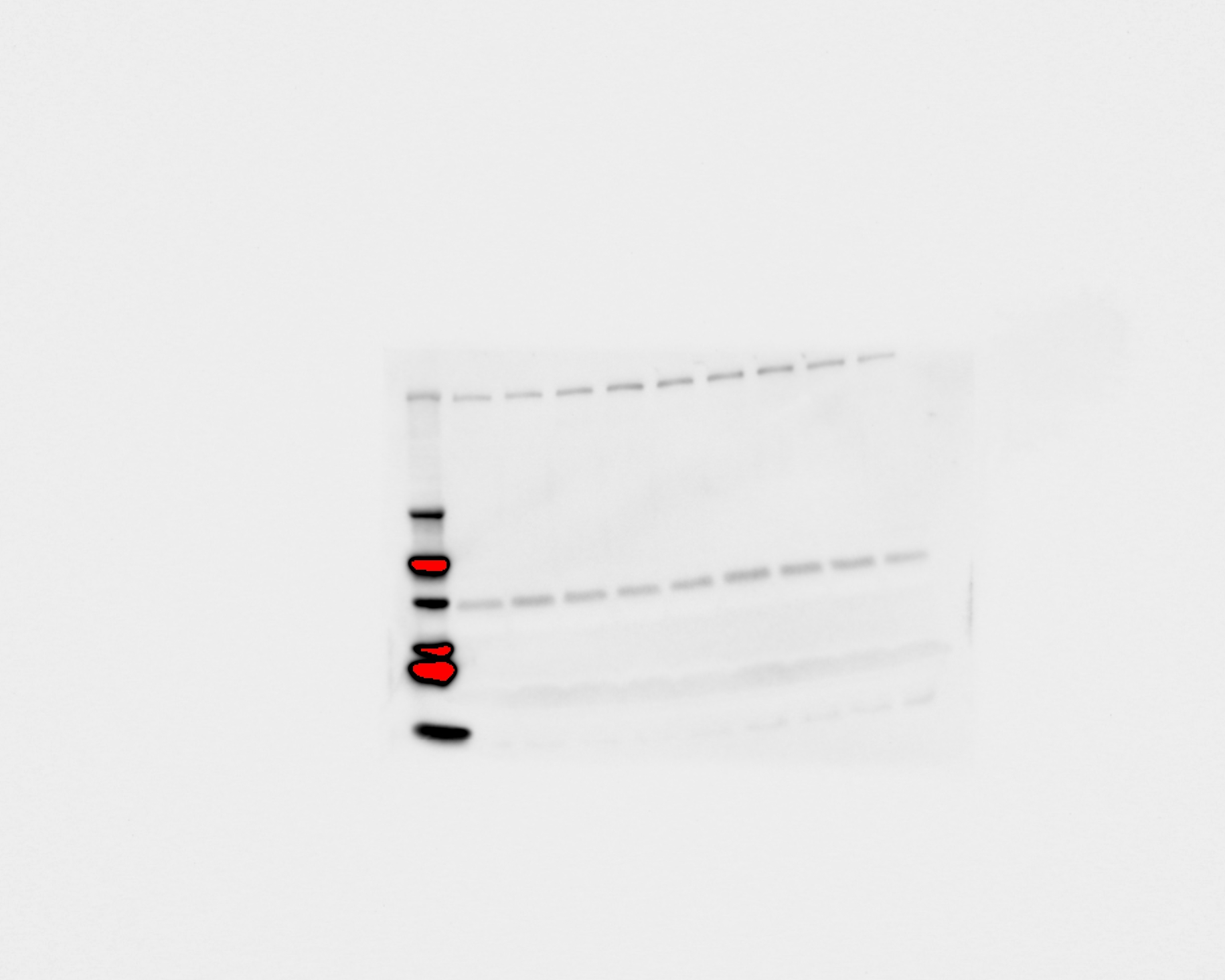

Supplement: Supplementary file 1 [file ijms-25-04129-s001.zip › IJMS WB/NBC.jpg]

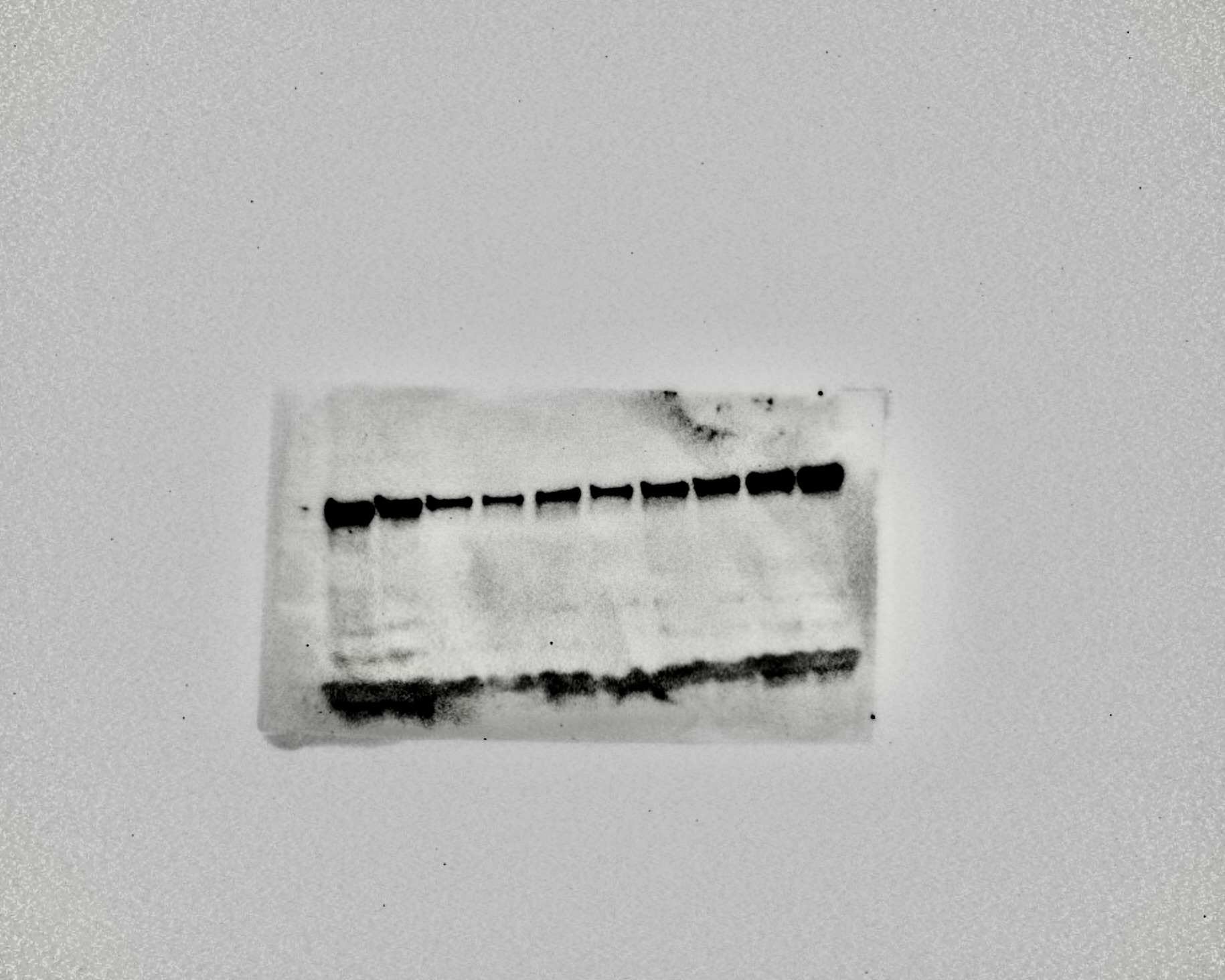

Supplement: Supplementary file 1 [file ijms-25-04129-s001.zip › IJMS WB/CS1.jpg]

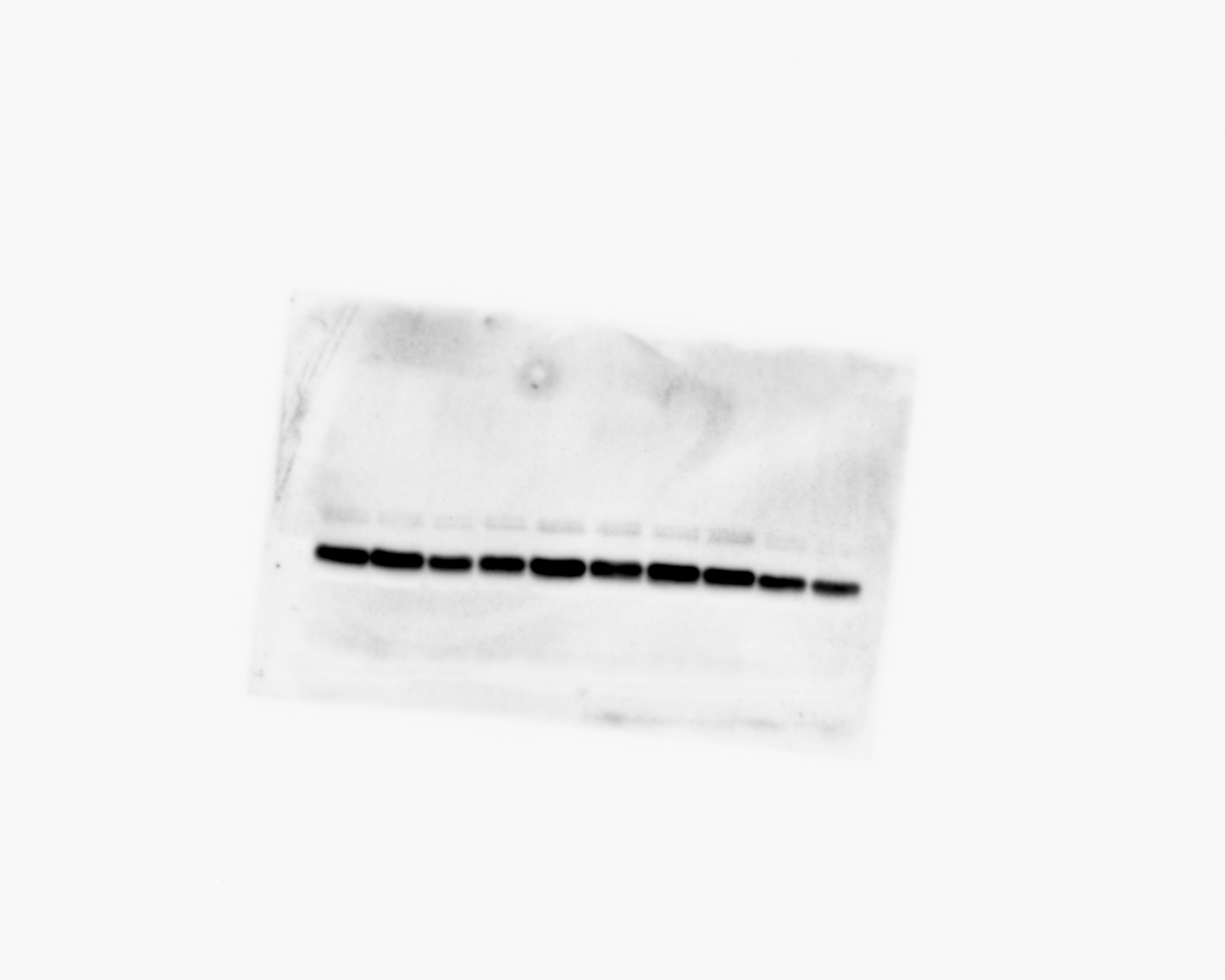

Supplement: Supplementary file 1 [file ijms-25-04129-s001.zip › IJMS WB/CS2.jpg]
